# Supplementary figures and images for: National differences in dissemination and use of open access literature
Source: PLoS One. 2022 Aug 9;17(8):e0272730. doi: 10.1371/journal.pone.0272730 (PMC9362937; doi:10.1371/journal.pone.0272730)

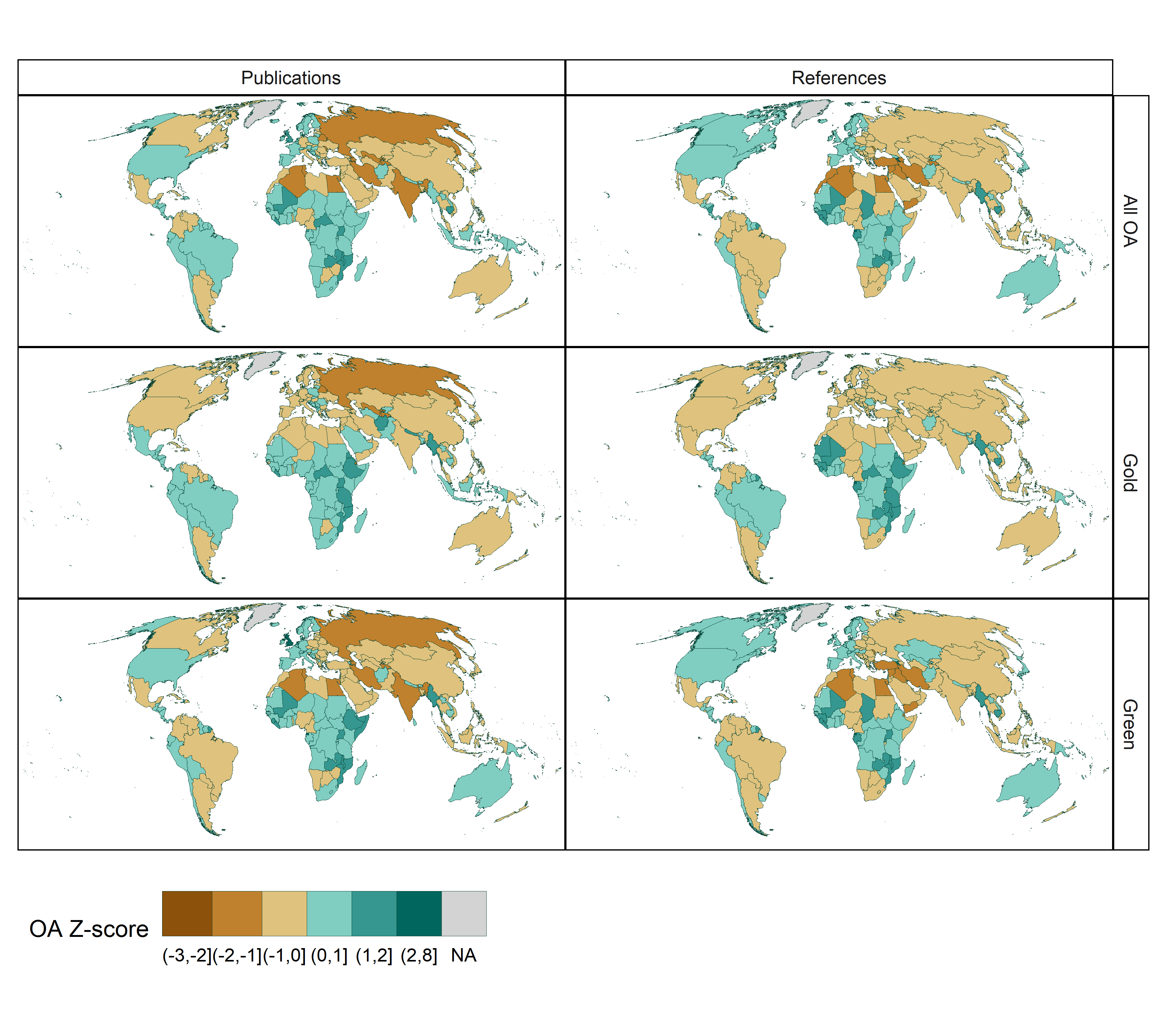

Supplement: S1 Appendix — Red indicates that a country is above the world average, blue indicates it is below the world average. White represents the world average. Contains information from OpenStreetMap and OpenStreetMap Foundation, which is made available under the Open Database License. (TIF) [file pone.0272730.s001.tif]
