# Supplementary material for: National differences in dissemination and use of open access literature
Source: PLoS One. 2022 Aug 9;17(8):e0272730. doi: 10.1371/journal.pone.0272730 (PMC9362937; doi:10.1371/journal.pone.0272730)

PLOS ONE

National differences in dissemination and use of open access literature

--Manuscript Draft--

**Manuscript Number:**	PONE-D-21-29770
**Article Type:**	Research Article
**Full Title:**	National differences in dissemination and use of open access literature
**Short Title:**	National differences in dissemination and use of open access literature
**Corresponding Author:**	Marc-André Simard<br>Université de Montréal: Universite de Montreal<br>Montréal, Quebec CANADA
**Keywords:**	open access; bibliometrics; open science; repositories
**Abstract:**	Open Access (OA) dissemination has been gaining a lot of momentum over the last decade, thanks to the implementation of several OA policies by funders and institutions, as well as the development of several new platforms that facilitate the publication of OA content at a low cost. Studies have shown that nearly half of the contemporary scientific literature could be available online for free. However, few studies have compared the use of OA literature across countries. This study aims to provide a global picture of OA adoption by countries, using two indicators: publications in OA and references made to articles in OA. We find that, on average, low-income countries are publishing and citing OA at the highest rate, while upper middle-income countries and higher-income countries publish and cite OA articles at below world-average rates. These results highlight national differences in OA uptake and suggest that more OA initiatives at the institutional, national, and international levels are needed to support a wider adoption of open scholarship.
**Order of Authors:**	Marc-André Simard
	Gita Ghiasi, Ph.D.
	Philippe Mongeon, Ph.D.
	Vincent Larivière, Ph.D.
**Additional Information:**	
**Question**	**Response**
**Financial Disclosure**<br><br>Enter a financial disclosure statement that describes the sources of funding for the work included in this submission. Review the submission guidelines for detailed requirements. View published research articles from *PLOS ONE* for specific examples.<br><br>This statement is required for submission and **will appear in the published article** if the submission is accepted. Please make sure it is accurate.	YES - Vincent Larivière would like you acknowledge the Canada Research Chair program (grant 950-231768). Marc-André Simard would like to acknowledge the SSHRC Joseph Armand Bombardier Master's Scholarship.

Powered by Editorial Manager® and ProduXion Manager® from Aries Systems Corporation

**Unfunded studies**<br>Enter: *The author(s) received no specific funding for this work.*<br><br>**Funded studies**<br>Enter a statement with the following details:<br>• Initials of the authors who received each award<br>• Grant numbers awarded to each author<br>• The full name of each funder<br>• URL of each funder website<br>• Did the sponsors or funders play any role in the study design, data collection and analysis, decision to publish, or preparation of the manuscript?<br>• **NO** - Include this sentence at the end of your statement: *The funders had no role in study design, data collection and analysis, decision to publish, or preparation of the manuscript.*<br>• **YES** - Specify the role(s) played.<br><br>* typeset	
**Competing Interests**<br><br>Use the instructions below to enter a competing interest statement for this submission. On behalf of all authors, disclose any competing interests that could be perceived to bias this work—acknowledging all financial support and any other relevant financial or non-financial competing interests.<br><br>This statement is required for submission and **will appear in the published article** if the submission is accepted. Please make sure it is accurate and that any funding sources listed in your Funding Information later in the submission form are also declared in your Financial Disclosure statement.<br><br>View published research articles from *PLOS ONE* for specific examples.	No

**NO authors have competing interests**<br><br>Enter: *The authors have declared that no competing interests exist.*<br><br>**Authors with competing interests**<br><br>Enter competing interest details beginning with this statement:<br><br>*I have read the journal's policy and the authors of this manuscript have the following competing interests: [insert competing interests here]*<br><br>* typeset	
**Ethics Statement**<br><br>Enter an ethics statement for this submission. This statement is required if the study involved:<br><br>• Human participants<br>• Human specimens or tissue<br>• Vertebrate animals or cephalopods<br>• Vertebrate embryos or tissues<br>• Field research<br><br>Write "N/A" if the submission does not require an ethics statement.<br><br>General guidance is provided below. Consult the submission guidelines for detailed instructions. **Make sure that all information entered here is included in the Methods section of the manuscript.**	N/A

**Format for specific study types**<br><br>**Human Subject Research (involving human participants and/or tissue)**<br>• Give the name of the institutional review board or ethics committee that approved the study<br>• Include the approval number and/or a statement indicating approval of this research<br>• Indicate the form of consent obtained (written/oral) or the reason that consent was not obtained (e.g. the data were analyzed anonymously)<br><br>**Animal Research (involving vertebrate animals, embryos or tissues)**<br>• Provide the name of the Institutional Animal Care and Use Committee (IACUC) or other relevant ethics board that reviewed the study protocol, and indicate whether they approved this research or granted a formal waiver of ethical approval<br>• Include an approval number if one was obtained<br>• If the study involved *non-human primates*, add *additional details* about animal welfare and steps taken to ameliorate suffering<br>• If anesthesia, euthanasia, or any kind of animal sacrifice is part of the study, include briefly which substances and/or methods were applied<br><br>**Field Research**<br><br>Include the following details if this study involves the collection of plant, animal, or other materials from a natural setting:<br>• Field permit number<br>• Name of the institution or relevant body that granted permission	
**Data Availability**<br><br>Authors are required to make all data underlying the findings described fully available, without restriction, and from the time of publication. PLOS allows rare exceptions to address legal and ethical concerns. See the PLOS Data Policy and FAQ for detailed information.	No - some restrictions will apply

<table>
<tr>
<td>
A Data Availability Statement describing where the data can be found is required at submission. Your answers to this question constitute the Data Availability Statement and will be published in the article, if accepted.

Important: Stating ‘data available on request from the author’ is not sufficient. If your data are only available upon request, select ‘No’ for the first question and explain your exceptional situation in the text box.

Do the authors confirm that all data underlying the findings described in their manuscript are fully available without restriction?
</td>
<td></td>
</tr>
<tr>
<td>
Describe where the data may be found in full sentences. If you are copying our sample text, replace any instances of XXX with the appropriate details.

If the data are held or will be held in a public repository, include URLs, accession numbers or DOIs. If this information will only be available after acceptance, indicate this by ticking the box below. For example: All XXX files are available from the XXX database (accession number(s) XXX, XXX.).
If the data are all contained within the manuscript and/or Supporting Information files, enter the following: All relevant data are within the manuscript and its Supporting Information files.
If neither of these applies but you are able to provide details of access elsewhere, with or without limitations, please do so. For example:

Data cannot be shared publicly because of [XXX]. Data are available from the XXX Institutional Data Access / Ethics Committee (contact via XXX) for researchers who meet the criteria for access to confidential data.

The data underlying the results presented in the study are available from (include the name of the third party

</td>
<td>
Data cannot be shared publicly because of copyrights with Clarivate Analytics. Data are available from the Web of Science data base. Open Access data are available for free on the Unpaywall database.
</td>
</tr>
</table>

and contact information or URL).<br>• This text is appropriate if the data are owned by a third party and authors do not have permission to share the data.<br><br>* typeset	
Additional data availability information:	

Manuscript

Click here to access/download;Manuscript;OA_draft_FINALV3.docx

National differences in dissemination and use of open access literature

Marc-André Simard[1], Gita Ghiasi[2], Philippe Mongeon[3], and Vincent Larivière[5]

1. École de bibliothéconomie et des sciences de l'information, Université de Montréal, Montréal, Qc, Canada
2. École de bibliothéconomie et des sciences de l'information, Université de Montréal, Montréal, Qc, Canada
3. School of Information Management, Dalhousie University, Halifax, NS (Canada)
4. École de bibliothéconomie et des sciences de l'information and Observatoire des sciences et des technologies, Université du Québec à Montréal, Montréal, Qc, Canada

Corresponding author:
Marc-André Simard
École de bibliothéconomie et des sciences de l'information
Université de Montréal
3150 Jean Brillant St, Montreal, Quebec, Canada
H3T 1N8
E-mail: marc-andre.simard.1@umontreal.ca

Word count: 6234
Tables: 2
Figures: 5
Appendix: 1
References: 45

Authors' contribution

Conceptualization: VL, MAS, PM, GG
Data Curation: PM
Formal Analysis: PM, MAS, GG
Funding acquisition: VL, MAS
Investigation: PM, MAS, GG
Methodology: PM, MAS, GG
Project Administration: MAS
Resources: VL
Software: PM, MAS, GG
Supervision: VL
Validation: PM
Visualization: MAS, GG, PM
Writing – Original Draft Preparation: MAS, PM, GG
Writing – Review & Editing: MAS, VL, PM, GG

Conflict of interest

The authors of this manuscript declare no conflict of interest.

Funding

Vincent Larivière would like you acknowledge the Canada Research Chair program (grant 950-231768). Marc-André Simard would like to acknowledge the SSHRC Joseph Armand Bombardier Master's Scholarship.

Abstract

Open Access (OA) dissemination has been gaining a lot of momentum over the last decade, thanks to the implementation of several OA policies by funders and institutions, as well as the development of several new platforms that facilitate the publication of OA content at a low cost. Studies have shown that nearly half of the contemporary scientific literature could be available online for free. However, few studies have compared the use of OA literature across countries. This study aims to provide a global picture of OA adoption by countries, using two indicators: publications in OA and references made to articles in OA. We find that, on average, low-income countries are publishing and citing OA at the highest rate, while upper middle-income countries and higher-income countries publish and cite OA articles at below world-average rates. These results highlight national differences in OA uptake and suggest that more OA initiatives at the institutional, national, and international levels are needed to support a wider adoption of open scholarship.

Introduction

Open Access (OA) dissemination makes research outputs freely available on the public internet, allowing users to read, download, copy, distribute, print, search, or link to the full text without any financial, legal, or technical barriers, in accordance with an open copyright licence **(1)**. One of its milestones of the widespread awareness for OA was 2001's Budapest Open Access Initiative, which established the first clear distinction between the two main types of OA: self-archiving (green OA) and a brand-new generation of journals that would allow scholarly material to be distributed in its full form, freely on the publisher's website (gold OA). Gold open access journals may be associated with article processing charges, originally intended to cover publication costs. However, the origins of OA go back to the 1970s, when computer scientists were already sharing their papers with File Transfer Protocol (FTP) servers. The first OA journals appeared in the late 1980s with *New Horizons in Adult Education*¸ *Psycholoquy*

(1989) and *Public-Access Computer systems Review* (1989). In the early 1990s, the movement had already picked up some steam with the creation of a dozen more journals, but more importantly, the creation of the first mainstream self-archiving repository *arXiv* (1991) by Paul Ginsparg, which allowed physics and engineering researchers to deposit preprint version of their papers for free on the internet. The year 1991 also marked the publication of Dr. Allen Bromley's, then advisor to President George H. W. Bush, *Policy Statements on Data Management for Global Change Research*. The statement pleaded for global open access, open data, and for the preservation of this data in open repositories **(2)**. In 2000, BioMed Central, the first for-profit open access publisher, was funded.

Nearly half a century since the first use of FTP servers to share scientific papers, the OA movement is still gaining momentum, with the implementation of several OA policies by funders and universities worldwide and the development of new business models by for-profit and non-for-profit publishers. According to several studies, nearly half of research articles could be available online at no cost **(3–5)**. Previous studies have analyzed the availability of articles **(3–5)**, their citation advantage **(4,6,7)**, and compliance to OA mandates **(8)**. It has often been argued that OA could improve global participation in science, especially for developing countries. However, very few articles **(9,10)** have compared how different countries, especially developing ones, use OA literature. This paper provides a contemporary portrait of the adoption of OA across the world, distinguishing between publishing in OA and citing OA publications, between the two main types of OA (green and gold).

Background

Open access models

This study is based on the original Budapest Open Access Initiative (2002) OA definition, which distinguishes between the two original models of OA: Gold Open Access and Green Open Access (or self-archiving). Gold journals are a type of scientific journals specifically committed to open access. They allow users to access their articles for free directly on their website. These journals may or may not charge article processing charges (APCs) to cover for the journal expenses. Ever since the early 2000s, the amount of gold OA journals in the Directory of Open Access Journals (DOAJ) has been growing exponentially, going from around 20 in 2002 to 16,621 as of July 17th, 2021. Indonesia has the highest number of OA journals with 1852, followed closely by the United Kingdom (1814) and Brazil (1637). Gold OA may sometimes also include the more recent model of hybrid open access which has been generally criticized by the scientific community for its so-called "double-dipping" practices: hybrid articles are published in subscription-based journals that offer the option of paying an additional fee, with publishers getting essentially paid twice for the same article (11,12).The bronze OA model has been recently introduced by Piwowar et al.(4). Bronze OA refers to publications that are openly directly on the journal or publisher's website but who do not have a clearly identifiable open license.

Self-archiving or green open access is achieved through the deposit of an article in an open electronic archive or repository. These electronic archives may be thematic (i.e. arXiv, *BioRxiv*), institutional (i.e. Harvard's *DASH*), or personal (i.e. a personal website). The access to repositories is generally free but may sometimes be delayed by an embargo pre-established by the article's publisher. Laakso (13) underlined the large gap between the potential self-archiving and the actual self-archiving done by researchers: over 80% of all scientific production, including 62% of articles published by the top 100 biggest publishers, could be available in OA based on publishers' self-archiving policies. However, other studies have

estimated that the current share of articles available in green OA is between 10 and 25% (4).There are currently 4,607 repositories listed in the Registry of Open Access Repositories (ROAR), including 1,021 in North America (839 in the United States,), 1702 in Europe, 179 in Africa, 1021 in Asia, and 584 in South America.

Several studies aimed to assess the number and percentage of scholarly papers that are freely available online. According to these different studies—which are based on different time periods, methodologies, and disciplines—between 20% and 54% of research articles are currently available online at no cost (3–5,14). Different aspects of the OA, such as the availability of articles (3–5,14,15), the so-called citation advantage of OA articles (4,6,7,13,16,17), and compliance to OA mandates (8,18,19), have also been addressed in the literature. According to a study by Piwowar et al. (4), 7.4% of a sample of 100,000 articles indexed by the Web of Science between 2009 and 2015 were available via gold OA, while 11.5% of the same articles were available on repositories. Other types of OA accounted for 17.2% of the sample, for a total of 36.1% of articles being available in OA. Using a sample of Unpaywall’s 2017 data, the same study found a proportion of 14.3% gold OA articles, 9.1% green OA articles, and 23% of articles available with other types of OA methods. One should note that in their study, Piwowar and her colleagues considered green and gold as mutually exclusive categories. A recent bibliometric study by Miguel et al. (20) showed that about 30% of a sample of 1179 articles about OA came from the United States, 13% from the United Kingdom, and 6% from Germany and Spain. However, a study by Basson et al. (21) underlined the importance of data sources and their effects of the measurement of open access, especially when assessing the global south where a large body of their scientific literature may not be indexed in the most popular bibliometric data sources such as Scopus or Web of Science.

A recent paper by Robinson-Garcia et al. (22) analyzed the OA uptake by universities worldwide, by combining data from Web of Science, Unpaywall and the Leiden Ranking disambiguation of 963 research institutions. Their results showed that the median share of OA publications of universities worldwide is 43%, with European universities generally leading the pack. In terms of OA usage by different at the country-level level (i.e., references to OA publications), a 2009 study by Evans and Reimer combined extensive bibliometric data with the World Bank and the UNESCO data on per capita gross national income (GNI) and found an overuse of closed articles in rich countries. However, their results also showed that the influence of OA was more than twice as strong in developing countries, but they noted that this influence was not as apparent in the very poorest countries where electronic access may be limited. They concluded that while the influence of OA is more modest than it has been proposed in the past, their results supported OA's potential to improve global participation in science. In the biomedical fields, Iyandemye and Thomas (10) showed the existence of a negative correlation between the GNI and the share of OA publications of a country, despite the low amount of local OA repositories available to researchers from developing countries.

OA mandates and policies have often been mentioned in the scientific literature as a possible way to encourage researchers and journals to publish in OA. An OA mandate is a policy adopted by a funding agency or a research institution that requires researchers to make their publications open access (11). Institutional mandates are generally less restrictive and tend to vary from an institution to another. Funding agency mandates are usually stricter: they impose a contractual obligation to publish in OA. Failure to comply may ultimately lead to a withholding of the funding. For instance, in the United States, the National Institute of Health's (NIH) public access policy (23) requires funded researchers to submit final peer-reviewed journal

manuscripts that arise from NIH funds to PubMed Central immediately upon acceptance for publication within the next 12 months after the date of publication. Failure to comply to this policy may lead to a suspension of the payment, although there is little evidence that they have done so in the past (8) OA mandates, especially those associated with Gold OA, have sometimes been criticized for restricting the freedom of researchers: they are only allowed to publish in gold open access journals, whereas green mandates are not very restrictive since most publishers already allow researchers to publish their papers into OA repositories (11). The Registry of Open Access Mandates and Policy (http://roarmap.eprints.org/) indexes OA mandates and policies adopted by universities, research institutions and funding agencies. According to ROARMAP, in the last fifteen years, the number of OA mandates increased from 123 in 2005 to 1,094 in 2021. The majority of OA mandates are currently in Europe (N=695), followed by the Americas (N=240), Asia (N=82), Oceania (N=42), and Africa (N=35). Although ROARMAP is a valuable tool to overview the state of open access policies worldwide, one must consider that information might be incomplete since it depends on voluntary registration by funding institutions.

Compliance to mandates vary from the type of mandates (institutional or funding) and across disciplines (8,24). Results obtained by Larivière and Sugimoto (8) have shown the positive effect of OA mandates on availability: out of 1.3 million articles subject to a mandate, around two thirds were available for free on the internet. Compliance ranges from 29% in some social sciences to 85% in biomedical research. Another study by Gargouri et al. (25) concluded that the stronger the OA mandate (more restrictive) is, the more researchers deposited a free version of the paper in repositories. On the other hand, Iyandemye and Thomas (10) showed a negative correlation between country per capita income and the percentage of open access publication in the biomedical fields, especially in sub-Saharan countries (despite the low number of

repositories and OA policies). They also found no clear relationship between the number of open access policies in a country and its percentage of open access publications.

Initiatives and Platforms

Over the last 20 years, several initiatives and platforms have been developed around the world to support OA dissemination. At the world level, UNESCO created the *Global Alliance of Open Access Scholarly Communication Platforms to Democratize Knowledge* to improve access to scholarly communications following a multicultural, multilingual, and multi-themed approach (26). UNESCO is also currently working on a Recommendation on Open Science that aims to define “shared values and principles for open science” and “identify concrete measures on Open Access and Open Data” (27). This recommendation is expected to be adopted by member states in November 2021. In Europe, Plan S, an ambitious project led by cOAlition S, aims to make every scientific article supported by public funds freely available on the internet by 2021 and remove or lower the article processing charges to low-income or middle-income countries (28). To illustrate the potential impact of Plan S on the scholarly communication infrastructure, in 2017, 35% of papers published in *Nature* and 31% of those in *Science* were funded by at least one of the coalition’s members (29).

In Canada, the non-for-profit platform Érudit is now the largest disseminator of French papers in North America. Today, more than 95% of its catalogue is available in OA (30). Another important Canadian initiative is the Public Knowledge Project which created the Open Journal Systems software (OJS). OJS is an open-source software that aims at simplifying the publication process for both publishers and researchers. There are currently 275 Canadian journals and 10,000 international journals using OJS (31). In the United States, there have been rumors of a new OA initiative comparable to Plan S in Europe: this policy would make every

publication funded by the federal government available online for free (32). A letter of response by the Association of American Publishers addressed to President Donald Trump has been signed by over 135 organizations from the scientific community, including the American Chemical Society, Elsevier, Wiley, and Wolters Kluwer (33). The letter states that a new OA policy would negatively affect innovation and peer review in science. Also in the United States, the PubMed Central repository was created in 2000 by the United States National Library of Medicine. In 2007, the National Institute of Health (NIH) introduced an OA policy (NIH Public Access Policy) which mandated that every article funded by the NIH had to be available for free within 12 months after publication (34), which has contributed to the expensive of PMC. Today, PMC (2021) indexes over 5.9 million papers, including over 8000 scientific journals.

In South America, the Scientific Electronic Library Online (SciELO) was created by the Brazilian government in 1997. It aims to provide the infrastructure needed to assist the publishing industry in developing countries and to give more visibility to scientific articles (35). As of today, SciELO indexes over 1,768 journals that have published over 874,000 articles in OA. In Asia, J-STAGE is an initiative funded by the government of Japan that aims to accelerate the dissemination of science by helping research institutions with the publication process. The J-STAGE database currently indexes over 5 million articles, including 4.7 million available in OA. In China, the Chinese Academy of Science (CAS) launched the China Open Access Journals (COAJ), which indexes OA journals from China. Since 2015, CAS has mandated its research institution to provide an OA version of their articles within 12 months of publication (36). In Africa, access to technologies, the internet and even to electricity remain a major problem in several countries, especially in Sub-Saharan Africa (Broadband Commission, 2019). In 1998, the International Network for the Availability of Scientific Publication created the African Journal OnLine (AJOL) project. It aims to improve dissemination and access to

research in Africa for African researchers and researchers worldwide. To this day, AJOL indexes over 525 journals (including 263 OA journals), and over 172 articles, with nearly two-thirds available in OA.

Research Objectives

The purpose of this paper is to provide a global picture of OA adoption by countries, using two indicators: publications in OA and references made to articles in OA. Our research questions are as follows:

1. What proportion of the scientific output of countries is OA?
2. To what extent are different countries citing literature in open access?
3. Are countries that disseminate more OA publications more likely to cite OA publications?
4. How is OA usage related to countries' level of income?

Methods

We collected all articles and reviews in the Web of Science (WoS) published between 2015 and 2019 (N=8,590,184). Science being increasingly collaborative, publications are generally authored by multiple individuals, often from different institutions and countries, among whom the work has typically been unevenly distributed (Larivière et al. 2015). We argue that choices in terms of cited literature and publication venues are predominantly made by authors who played a leading role in the research. Thus, when assigning a publication to a country, we only use the institutional affiliations of the first and corresponding authors. We further classified countries based on the World Bank's country classifications by income level (2021–2022 ;

https://blogs.worldbank.org/opendata/new-world-bank-country-classifications-income-level-2021-2022).

We used the Unpaywall (http://unpaywall.org) database to determine the OA availability and OA type of a paper or reference. Because we use the DOI to link the WoS and Unpaywall data, the 381,101 WoS publications that do not include a DOI, and the 71,408 dois that were not found in Unpaywall were dropped, leaving us with a dataset of 8,137,675 publications. This data indicates whether the paper is published in OA or not, and in the OA category it belongs to (gold, green, hybrid or bronze). Some of our analyses include all OA categories while others were limited to green and gold to stay true to the original BOAI definition. These two categories are not mutually exclusive and papers that are published in a gold OA journal and found in a repository were counted in both categories.

The percentage of OA publication varies drastically across disciplines (37) and ~~that~~ the distribution of publications across fields varies by country. To account for this, we calculate the percentage of gold and green OA publications for each country and field, and then calculate the field-weighted average z-score for each country. These z-scores are used for all the country-level analyses below.

Results

Tables 1 and 2 presents an overview of our dataset by discipline and type of OA in terms of publications and references, respectively. In table 1, we see that 42.9% of the articles published during the 2015-2019 period were Open Access at the time of our data collection. The proportion of publications in OA varies by fields, ranging from 21.2% in the Humanities to

50% in the Medical and Health Sciences. The very small proportion of papers that are in gold OA only (0.5% - 6.1%) shows the large overlap between green and gold OA.

Table 1. Proportion of OA publications by type and by fields (2015-2019.

Field	*N*	*OA*	*Gold*	*Green*	*Gold Only*	*Green Only*
Natural Sciences	4,282,099	45.4	19.9	36.3	3.7	20.1
Engineering and Technology	2,307,970	30.4	13.0	21.4	4.9	13.3
Medical and Health Sciences	2,634,315	50.0	20.8	40.4	2.0	21.6
Agricultural Sciences	402,115	35.9	17.1	22.0	6.1	11.0
Social Sciences	795,087	35.5	7.9	29.8	1.9	23.8
Humanities	266,985	21.2	5.9	15.8	2.6	12.5
Unknown	33,666	35.8	2.2	31.3	0.5	29.6
All Fields	8,590,184	42.9	18.1	33.8	3.4	19.1

In table 2, we see that the share of references to OA papers also varies by fields with Medical and Health Sciences citing more OA papers. However, we can observe that the proportion of references to gold OA articles (7.2%) is generally lower than the proportion of gold OA publications presented in table 1 (18.1%). Overall, the proportion of OA references is lower than the proportion of OA publications.

Table 2. Proportion references to OA by type and by fields.

Field	*Number of references*	*OA*	*Gold*	*Green*	*Gold Only*	*Green Only*
Natural Sciences	150,284,096	40.9	7.5	32.4	0.7	14.8

Engineering and Technology	66,541,412	25.9	5.0	20.6	0.8	11.9
Medical and Health Sciences	86,728,869	50.6	9.2	37.1	0.6	14.2
Agricultural Sciences	11,536,124	33.4	8.1	22.2	1.2	7.7
Social Sciences	21,145,993	32.7	3.3	28.3	0.2	20.2
Humanities	1,785,285	27.0	2.8	23.1	0.3	16.2
Unknown	900,121	39.9	4.9	36.2	1.1	27.8
All Fields	338,921,900	39.6	7.2	30.6	0.7	14.2

Figures 1 and 2 reveal that countries mostly cite OA more often than they publish in OA. Sub-Saharan African countries publish and use OA more often. In North America, the United States publishes and cites OA more than the world average, however, Canada publishes in OA less often but cites OA more often. South American countries are generally slightly over the average, with western countries such as Ecuador, Peru, and Chile generally publishing and citing OA more, while Brazil is at the world average. In Europe, Western European countries mostly publish and cite OA, while the trend is opposite for the Eastern European countries. In Asia, North Africa and the Middle East, most countries publish or cite OA less often.

Figure 1a. Figure 1b.

Figure 1. Weighted z-score and normalized map of the number of OA publications by country. Red indicates that a country is above the world average, blue indicates it is below the world average. White represents the world average.

Figure 2a. Figure 2b.

Figure 2. Weighted z-score and normalized map of references made to OA publications by country. Red indicates that a country above the world average, blue indicates it is below the world average. White represents the world average.

Figure 3 illustrates the relationship between the weighted and normalized OA publication indicator of countries with the weighted and normalized reference indicator. A Pearson

correlation shows a moderate relationship between the two indicators ($r = 0.4805$; $p<0.001$), which indicates that the more a country publishes in OA, the more it is generally likely to make references to OA papers.

Figure 3.

Figure 3. Correlation between normalized OA publications and normalized cited references, by country.

Figure 4 shows plots of countries according to their OA publications and references compared to the field-normalized world average. We observe that overall, as figure 1 suggested, the two indicators correlate. The correlation is, however, much stronger for low-income ($r=0.884$; $p<0.001$) and lower-middle-income countries ($r=0,827$; $p<0.001$) than for upper-middle-income countries ($r=0.524$; $p<0.001$) and high-income countries ($r=0.630$; $p<0.001$). On average, the different groups are positioned in different quadrants: the high-income countries are mostly positioned in the first and third quadrants, while upper-middle-income countries are in the third quadrant. On the other hand, low-income and lower-middle-income countries are generally located in the first quadrant. This shows that overall, lower income countries tend to both publish and cite OA more than the rest of the world, while the upper-middle-income countries are generally publishing and citing OA under the world average. Open access practices tend to vary a lot from a country to another in high-income countries with about half being over the average and the other half being under.

Figure 4.

Figure 4. OA publication vs. references per country income (red dots; average for all the countries of the same income category).

Figure 5 shows the average weighted z-score for green and gold OA publications, and references to green and gold OA publication by income categories. It shows that while low-income countries tend to publish and cite both green and gold OA, high-income countries

mostly use green OA, while considerably underusing gold OA. Looking at middle-income countries, lower-middle-income countries tend to use gold OA more than green OA, while upper-middle-income countries underuse both types of OA. Appendix 1 shows detailed world maps of gold and green OA publications and reference patterns.

Figure 5.

Figure 5. Average weighted z-score for green and gold OA publications (left) and references to green and gold OA publications (right) by income category.

Conclusion

Our findings show that for the period between 2015 and 2019, Sub-Saharan Africa is publishing and citing OA at a higher rate than the rest of the world, while we found that the Middle East and Asia are the areas where the proportion of publications available in OA is lower, as is their use of OA. This could be explained by the fact that APCs are mostly waived for Sub-Saharan countries (e.g. research4life countries) but are not or only partially waived for most Middle Eastern and Asian countries. One other possible explanation is that the national and transnational OA initiatives such as plan S for the European Union and PubMed Central in the United States are almost non-existent in these countries, and the Registry of Open Access Repositories shows that institutional repositories are not well developed. Looking at income levels, our findings also reveal that lower middle-income and low-income countries are those who publish and cite the most OA. Again, this may be partly explained by the fact that APCs are generally waived for these countries, making publishing in gold OA more accessible for them. Moreover, some commercially owned gold OA journals may have less strict publication criteria and given the lack of research infrastructure in these countries, they may be more likely to publish in these journals (38). We also found that the upper-middle-income countries tend to

behave similarly to the higher-income countries. However, the underlying mechanisms behind the use of OA potential may be different. For instance, upper middle-income countries may lack the resources to pay for APCs on top of high subscription prices for closed journals (both of which they don't qualify for waivers), and which is not necessarily the case for high-income countries. There may also be other factors, such as a lower reputation of OA journals in certain parts of the world. Another interesting finding is that while low-income countries use both green and gold OA, high-income countries generally tend to favour green OA. This may imply that even in the richest countries on the planet, researchers may still struggle to pay APCs. Overall, our results seem to confirm previous studies (9,10) which both found that low income countries tend to publish in OA more than the other countries, despite the fact that the former having been conducted more than a decade ago and the latter only assessing biomedical research. Ultimately, while high-income countries have mandates and repositories, and low-income countries have waivers, our results highlight national differences in OA uptake and suggest that more OA initiatives at the institutional, national, and international levels are needed to support a wider adoption of open scholarship.

Our study has provided a snapshot of the state of OA over the 2015-2019 period. While a significant share of the scientific output was available in open access at the time of this study, concerns have been raised about the preservation of open access literature and the integrity of the scholarly communication record (39). Combining several bibliographic indexes such as Scopus, Ulrichsweb, and the Directory of Open Access Journals, the authors traced journals through the Internet Archive's Wayback Machine. Their results showed that 174 OA journals from various disciplines and geographic regions had completely disappeared from the web between 2000 and 2019. While this number does not appear to be particularly high, the authors noted that their results were most likely a low estimation since the limitations in the availability

of data did not allow them to assess the full extent of the phenomenon. They also noticed some common traits between the vanished journals: they tended to be affiliated with academic institutions or scholarly societies, located in North America, or published social sciences and humanities research. Ultimately, their study underlined the importance of collaborative action to ensure continued access and to prevent the loss of further scholarly knowledge. It remains uncertain how this may play a part in the general uptake and sustainability of OA. Further research on this topic is certainly needed.

The recent COVID-19 pandemic has underlined the importance of OA, which has proven essential for a faster and more efficient dissemination and use of the scholarly literature. At the journal level, several publishers such as Springer-Nature, AAAS (Science), Elsevier, and Massachusetts Medical Society (NEJM) have announced the free opening of their COVID-19 related papers. However, it remains unclear if these papers will remain open access, as some publishers such as Elsevier have already mentioned that their Novel Coronavirus Information Center will only be free for the duration of the pandemic (40). Despite the existence of geological tensions (41–43) and initiatives such as China's move to ensure that funded studies on COVID-19 will be published in Chinese journals over international journals (44), the majority of countries have increased their number of international collaborations and have generally increased their share of OA literature (43,45). However, it has been shown that countries' previous OA dissemination practices had no significant effect on their OA publication practices during the ongoing global pandemic: the extent to which a country was affected by COVID-19 was the main factor for OA publication (43). Another important finding from Lee and Haupt study is that countries with a higher GDP may not engage as much as other countries in international collaboration and open access during the global crisis, most likely because they are less dependent on outside collaborators. These findings have several

implications for our study. First, the pandemic may help middle-income countries to participate and alleviate the divide in the uptake of OA since they do not necessarily have the necessary funding and resources to produce national COVID-19 research. Second, with high-income countries being more reluctant to participate in global collaboration and publish their COVID-19 research in OA, it highlights the importance of the various OA mandates, policies, and repositories in place. It remains to be seen whether this recent trend created by the pandemic will persist over time. Ultimately, the current crisis shows the necessity for more sustainable OA dissemination channels: as long as the research community is dependent on private for-profit publishers, the "openness" of articles will depend on either the "good will" of those publishers or on the scientific community's capacity and willingness to pay for it.

References

1. Budapest Open Access Initiative. Read the Budapest Open Access Initiative [Internet]. 2002 [cited 2021 Jul 21]. Available from: https://www.budapestopenaccessinitiative.org/read

2. Bromley A. Policy Statements on Data Management for Global Change Research [Internet]. 1991. Available from: http://www.gcrio.org/USGCRP/DataPolicy.html

3. Archambault E, Amyot D, Deschamps P, Nicol A, Provencher F, Rebout L, et al. Proportion of Open Access Papers Published in Peer-Reviewed Journals at the European and World Level —1996–2013 [Internet]. European Commission; Science-Metrix; 2014. Report No.: RTD-B6-PP-2011-2. Available from: https://science-metrix.com/sites/default/files/science-metrix/publications/d_1.8_sm_ec_dg-rtd_proportion_oa_1996-2013_v11p.pdf

4. Piwowar H, Priem J, Larivière V, Alperin JP, Matthias L, Norlander B, et al. The state of OA: a large-scale analysis of the prevalence and impact of Open Access articles. PeerJ. 2018 Feb 13;6:e4375.

5. European Commission. Trends for open access to publications [Internet]. 2019 [cited 2021 Jul 21]. Available from: https://ec.europa.eu/info/research-and-innovation/strategy/goals-research-and-innovation-policy/open-science/open-science-monitor/trends-open-access-publications_en

6. Antelman K. Do Open-Access Articles Have a Greater Research Impact? Coll Res Libr. 2004 Sep 1;65(5):372–82.

7. Swan A. The Open Access citation advantage: Studies and results to date. Univ Southampt Institutional Repos [Internet]. 2010; Available from: https://eprints.soton.ac.uk/268516/

8. Larivière V, Sugimoto CR. Do authors comply when funders enforce open access to research? Nature. 2018 Oct;562(7728):483–6.

9. Evans JA, Reimer J. Open Access and Global Participation in Science. Science. 2009 Feb 20;323(5917):1025–1025.

10. Iyandemye J, Thomas MP. Low income countries have the highest percentages of open access publication: A systematic computational analysis of the biomedical literature. Glanzel W, editor. PLOS ONE. 2019 Jul 29;14(7):e0220229.

11. Suber P. Open access. Cambridge, Mass: MIT Press; 2012. 242 p. (MIT Press essential knowledge series).

12. Pinfield S, Salter J, Bath PA. The “total cost of publication” in a hybrid open-access environment: Institutional approaches to funding journal article-processing charges in combination with subscriptions. J Assoc Inf Sci Technol. 2016 Jul;67(7):1751–66.

13. Laakso M. Green open access policies of scholarly journal publishers: a study of what, when, and where self-archiving is allowed. Scientometrics. 2014 May;99(2):475–94.

14. Laakso M, Björk B-C. Hybrid open access—A longitudinal study. J Informetr. 2016 Nov;10(4):919–32.

15. Archambault E, Amyot D, Deschamps P, Nicol A, Provencher F, Rebout L, et al. Proportion of Open Access Peer-Reviewed Papers at the European and World Levels—2004-2011 [Internet]. European Commission; Science-Metrix; 2013. Report No.: RTD-B6-PP-2011-2. Available from: science-metrix.com/pdf/SM_EC_OA_Availability_2004-2011.pdf

16. Hajjem C, Harnad S, Gingras Y. Ten-Year Cross-Disciplinary Comparison of the Growth of Open Access and How it Increases Research Citation Impact. arXiv:cs/0606079 [Internet]. 2006 Aug 15 [cited 2021 Sep 13]; Available from: http://arxiv.org/abs/cs/0606079

17. McCabe MJ, Snyder CM. IDENTIFYING THE EFFECT OF OPEN ACCESS ON CITATIONS USING A PANEL OF SCIENCE JOURNALS. Econ Inq. 2014 Oct;52(4):1284–300.

18. Anne Kennan M. Learning to share: mandates and open access. Libr Manag. 2011 May 17;32(4/5):302–18.

19. Harnad S, Brody T, Vallières F, Carr L, Hitchcock S, Gingras Y, et al. The Access/Impact Problem and the Green and Gold Roads to Open Access: An Update. Ser Rev. 2008 Mar;34(1):36–40.

20. Miguel S, Tannuri de Oliveira E, Cabrini Grácio M. Scientific Production on Open Access: A Worldwide Bibliometric Analysis in the Academic and Scientific Context. Publications. 2016 Jan 14;4(1):1.

21. Basson I, Simard M-A, Aubierge Z, Sugimoto CR, Lariviere V. Data sources and their effects on the measurement of open access. Comparing Dimensions with the Web of Science. Proc 18th Int Conf Scientometr Informetr. 2021;93–8.

22. Robinson-Garcia N, Costas R, van Leeuwen TN. Open Access uptake by universities worldwide. PeerJ. 2020 Jul 8;8:e9410.

23. National Institutes of Health. NIH Public Access Policy [Internet]. 2021 [cited 2021 Sep 13]. Available from: https://publicaccess.nih.gov/

24. Vincent-Lamarre P, Boivin J, Gargouri Y, Larivière V, Harnad S. Estimating open access mandate effectiveness: The MELIBEA score. J Assoc Inf Sci Technol. 2016 Nov;67(11):2815–28.

25. Gargouri Y, Hajjem C, Larivière V, Gingras Y, Carr L, Brody T, et al. Self-Selected or Mandated, Open Access Increases Citation Impact for Higher Quality Research. Futrelle RP, editor. PLoS ONE. 2010 Oct 18;5(10):e13636.

26. UNESCO. Launch of the Global Alliance of Open Access Scholarly Communication Platforms to democratize knowledge [Internet]. 2019 [cited 2021 Jul 16]. Available from: https://en.unesco.org/news/launch-global-alliance-open-access-scholarly-communication-platforms-democratize-knowledge.

27. UNESCO. Towards a UNESCO Recommendation on Open Science [Internet]. 2020 [cited 2021 Jul 16]. Available from: https://en.unesco.org/sites/default/files/open_science_brochure_en.pdf

28. cOAlition S. Guidance on the implementation of Plan S [Internet]. cOAlition S. 2018 [cited 2021 Jul 15]. Available from: https://www.coalition-s.org/wp-content/uploads/271118_cOAlitionS_Guidance.pdf

29. Brainard J. Open access takes flight. Science. 2021 Jan 1;371(6524):16–20.

30. Érudit. Érudit [Internet]. 2021 [cited 2021 Jul 21]. Available from: https://erudit.org

31. Stranack K. How Many Journals Use OJS? [Internet]. Public Knowledge Project. 2015 [cited 2021 Jul 15]. Available from: https://pkp.sfu.ca/2015/10/01/how-many-journals-use-ojs/

32. Subbaraman N. Rumours fly about changes to US government open-access policy. Nature. 2019 Dec 20;d41586-019-03926–1.

33. Association of American Publishers. Letter to the President [Internet]. 2020. Available from: https://presspage-production-content.s3.amazonaws.com/uploads/1508/lettertothepresidentfrom140researchandpublishingorg2.pdf?10000.

34. Lowey NM. H.R.2764—Consolidated Appropriations Act, 2008 [Internet]. 2764 2008. Available from: http://congress.gov/bill/110th-congress/house-bill/2764/text/enr

35. Packer AL. SciELO - A model for cooperative electronic publishing in developing countries. In: Redefining the Information Chain - New Ways and Voices: Proceedings of an ICCC/IFI Conference [Internet]. Karlskrona/Ronneby, Sweden: ELPUB; 1999. Available from: https://elpub.architexturez.net/doc/oai-elpub-id-9927

36. Chen E. 5 Tips for Understanding STM Open Access in China. [Internet]. Copyright Clearance Center. 2018 [cited 2021 Jul 15]. Available from: http://copyright.com/blog/5-tips-understanding-stm-open-access-china/

37. Maddi A. Measuring open access publications: a novel normalized open access indicator. Scientometrics. 2020 Jul;124(1):379–98.

38. Butler D. Investigating journals: The dark side of publishing. Nature. 2013 Mar;495(7442):433–5.

39. Laakso M, Matthias L, Jahn N. Open is not forever: A study of vanished open access journals. J Assoc Inf Sci Technol. 2021 Sep;72(9):1099–112.

40. Elsevier. Novel Coronavirus Information Centre [Internet]. 2021. Available from: https://www.elsevier.com/connect/coronavirus-information-center

41. Crowley M, Wong E, Jakes L. Coronavirus drives the US and China deeper into global power struggle. New York Times [Internet]. 2020; Available from: https://www.nytimes. com/2020/03/22/us/politics/coronavirus-us-china.html

42. Mercer P. Australia-China tensions over call for global COVID-19 probe. Voice of America. VOA News [Internet]. 2020; Available from: https://www.voanews.com/covid-19-pandemic/australia-china-tensions-over-call-global-covid-19-probe

43. Lee JJ, Haupt JP. Scientific Collaboration on COVID-19 Amidst Geopolitical Tensions between the US and China. J High Educ. 2021 Feb 23;92(2):303–29.

44. Lariviere V, Shu F, Sugimoto CR. The Coronavirus (COVID-19) outbreak highlights serious deficiencies in scholarly communication. 2020; Available from: https://blogs.lse.ac.uk/impactofsocialsciences/2020/03/05/the-coronavirus-covid-19-outbreak-highlights-serious-deficiencies-in-scholarly-communication/

45. Torres-Salinas D, Robinson-Garcia N, Castillo-Valdivieso PA. Open Access and Altmetrics in the pandemic age: Forescast analysis on COVID-19 literature [Internet]. Scientific Communication and Education; 2020 Apr [cited 2021 Sep 13]. Available from: http://biorxiv.org/lookup/doi/10.1101/2020.04.23.057307

Acknowledgements

The authors would like to thank Vanessa Sandoval-Romero for her participation in the initial phases of the project. This article is a revised version of an ISSI paper with updated data that was originally published in the Proceedings of the 18th International Conference on Scientometrics and Informetrics.

Appendix

Figure 6. Weighted z-score and normalized map of the number of OA publications and references made to OA publications by country. Red indicates that a country is above the world average, blue indicates it is below the world average. White represents the world average.

Figure 6a.

Figure 6b.

Figure 1a

Click here to access/download;Figure;fig1a.tif

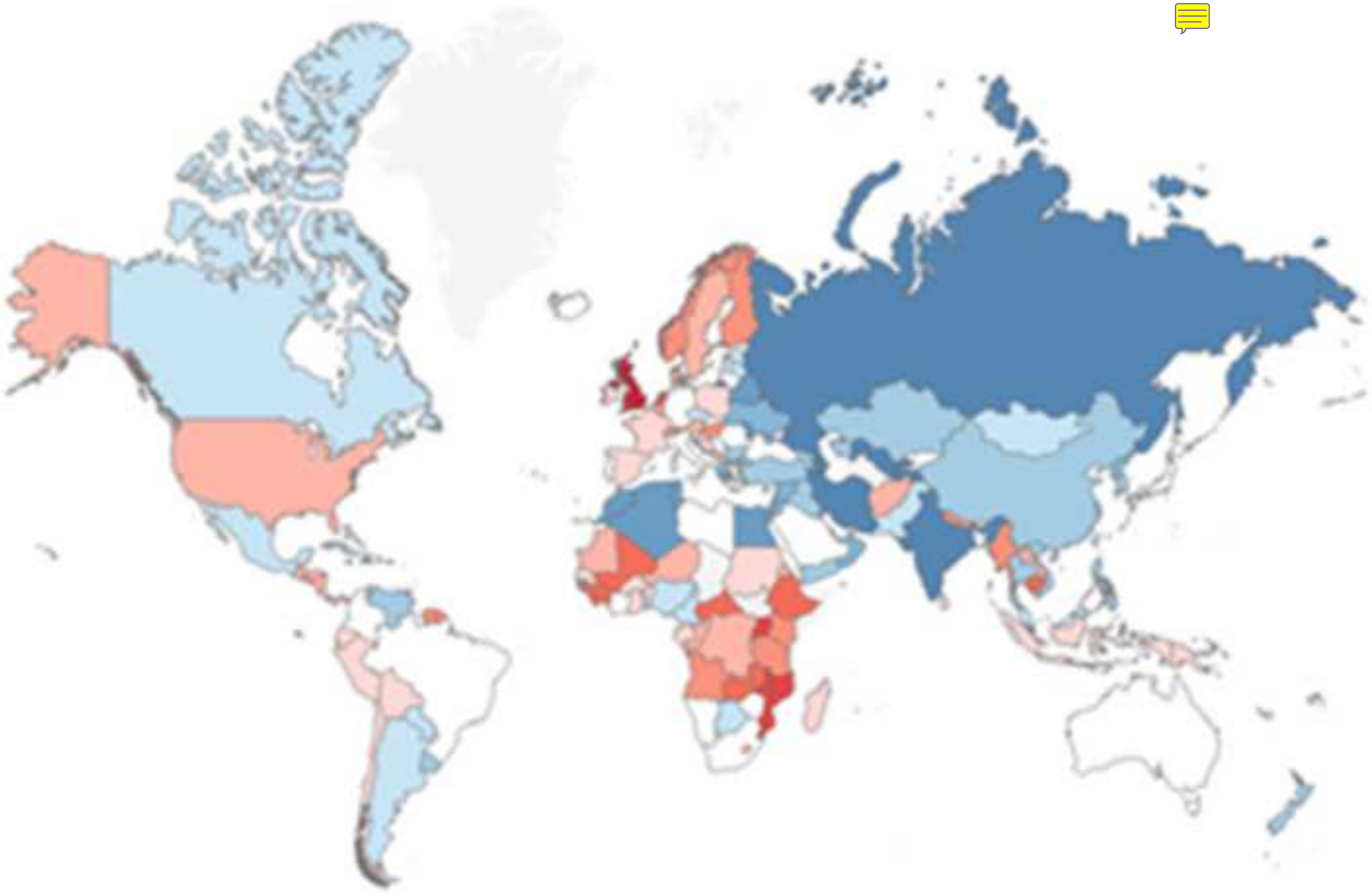

Figure 1b

Click here to access/download;Figure;fig1b.tif

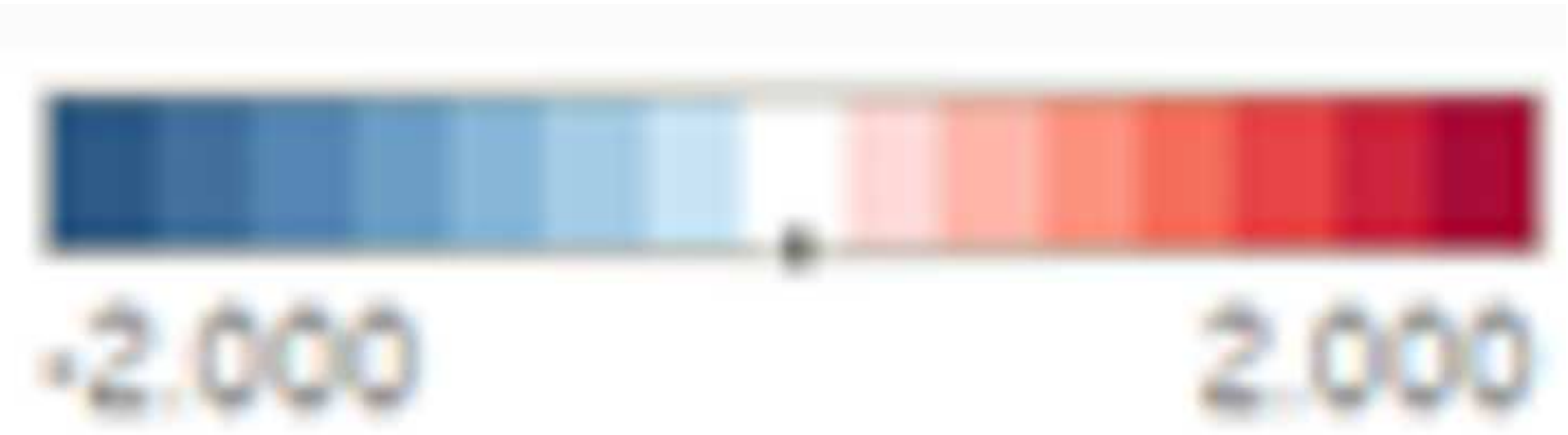

Figure 2a

Click here to access/download;Figure;fig2a.tif

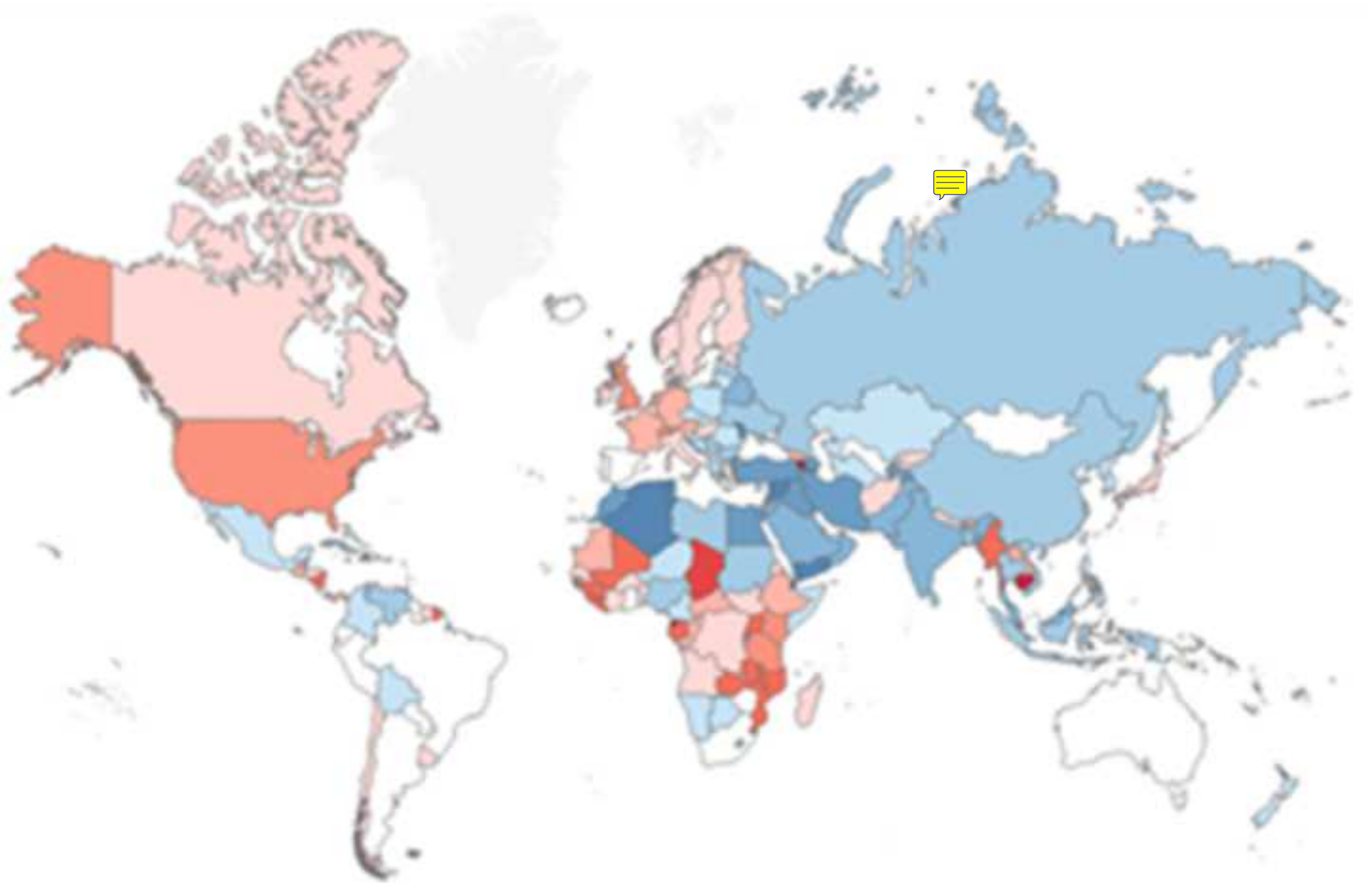

Figure 2b

Click here to access/download;Figure;fig2b.tif

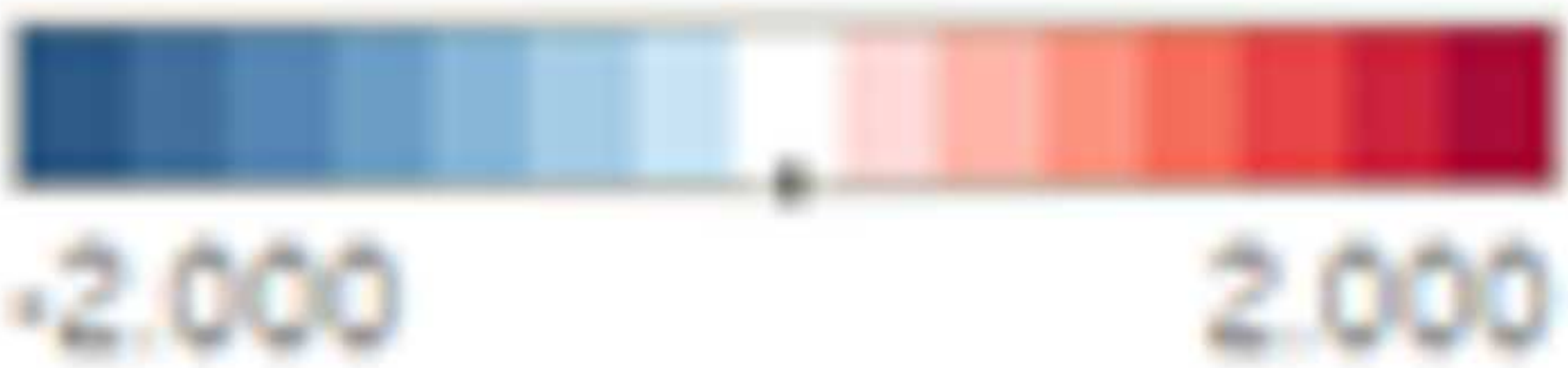

Figure 3

Click here to access/download;Figure;fig3.tif

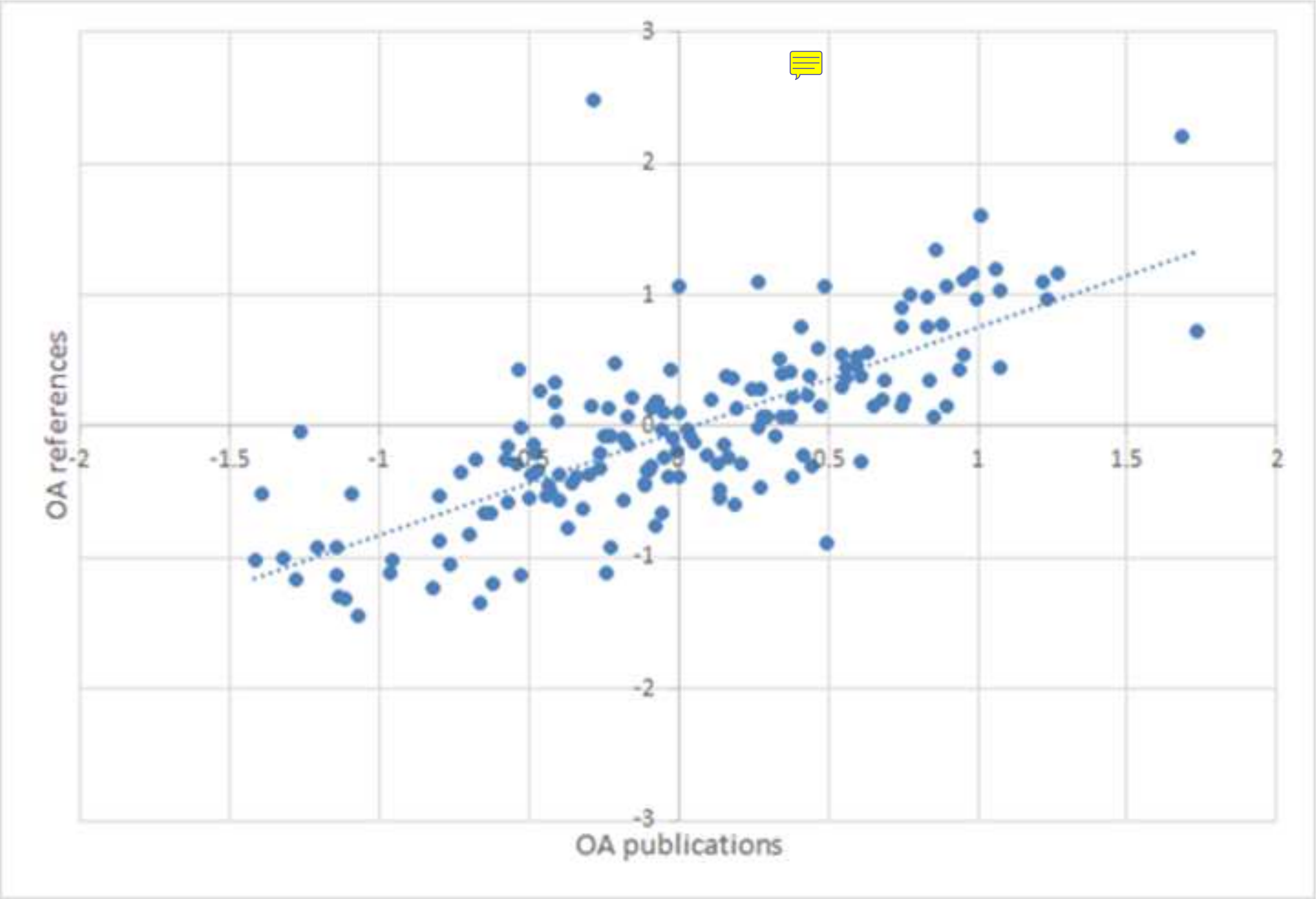

Figure 4

Click here to access/download;Figure;fig4.tif

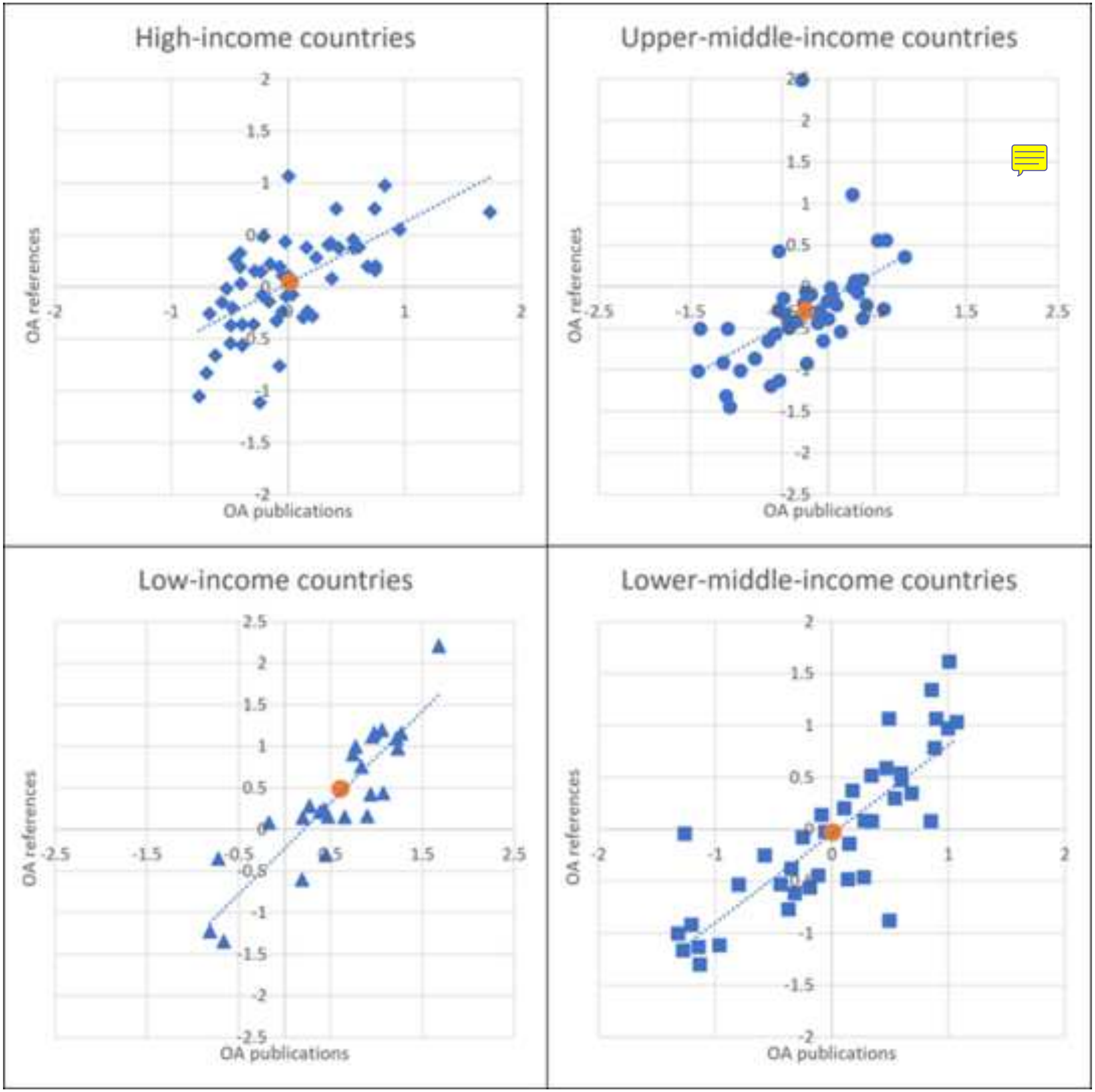

Figure 5

Click here to access/download;Figure;fig5.tif

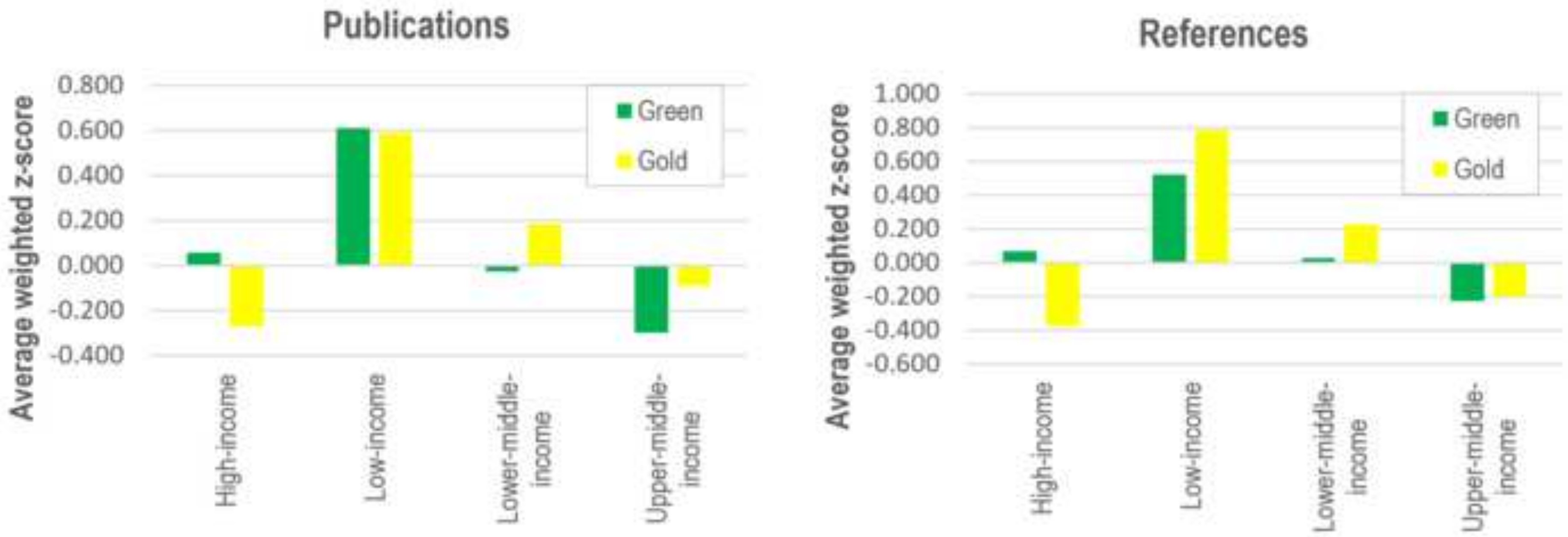

Appendix 1a

Click here to access/download;Figure;appendix1a.tif

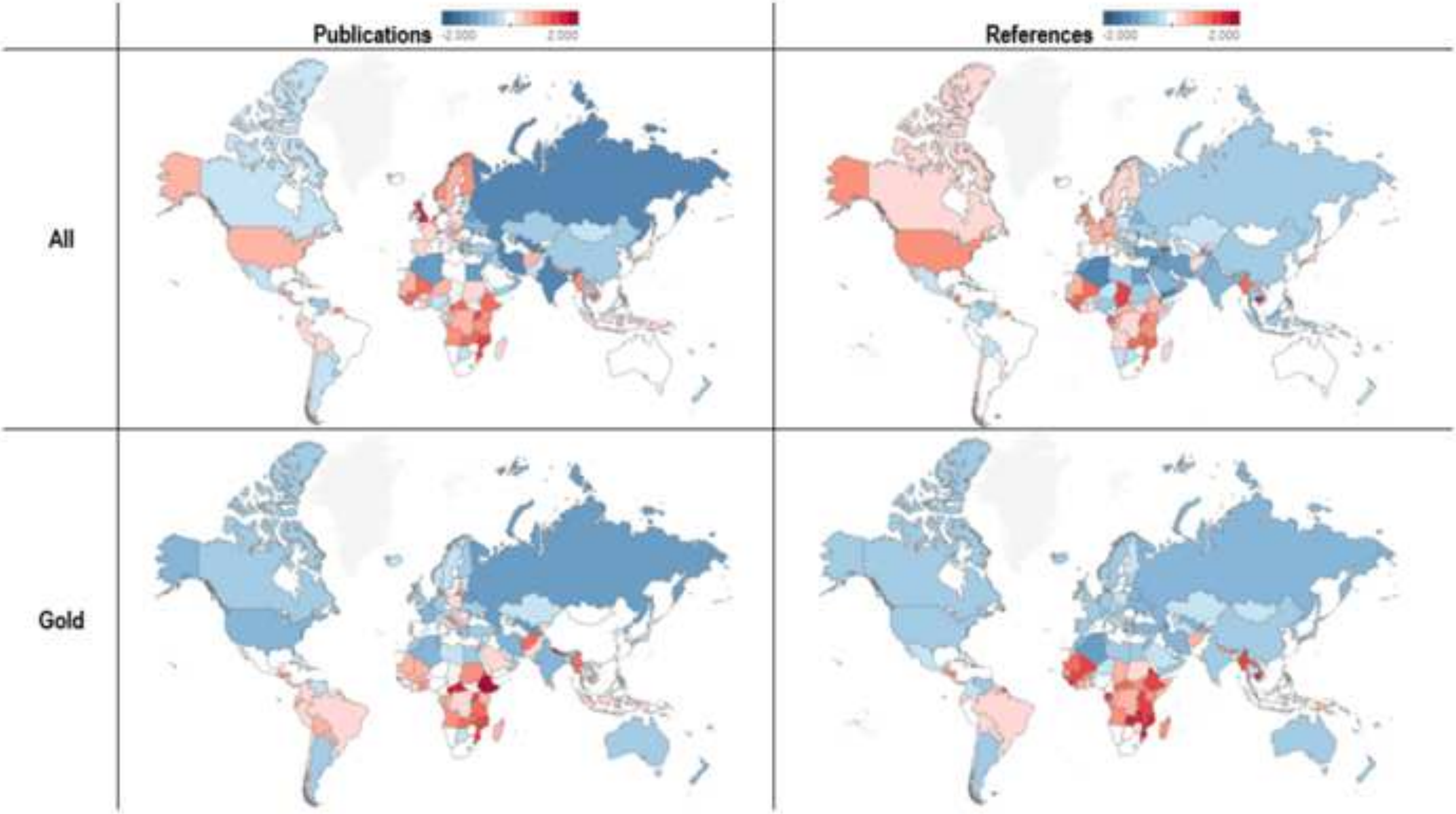

Appendix 1b

Click here to access/download;Figure;appendix1b.tif

Green

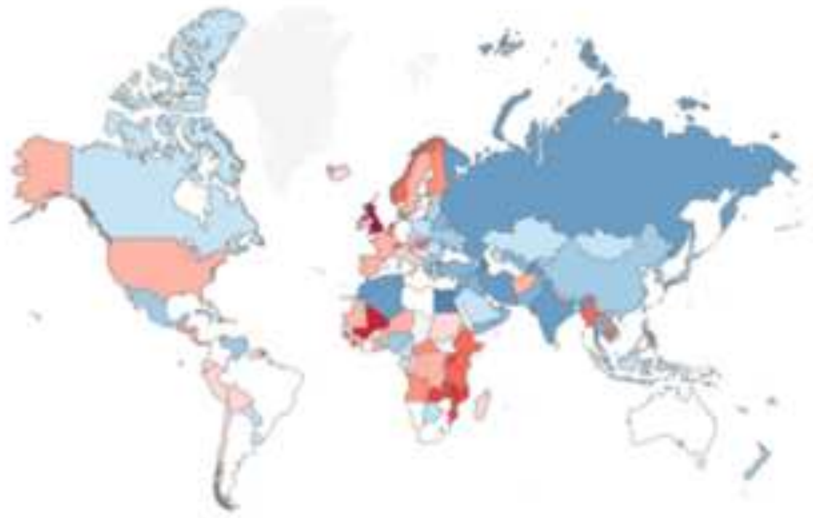

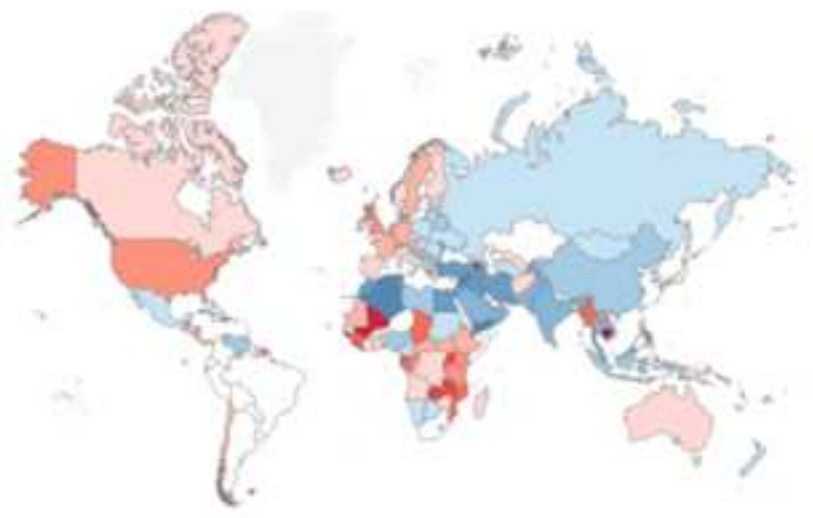

Supplement: Attachment — Submitted filename: PONE-D-21-29770_reviewer.pdf [file pone.0272730.s002.pdf]
