# Supplementary material for: National differences in dissemination and use of open access literature
Source: PLoS One. 2022 Aug 9;17(8):e0272730. doi: 10.1371/journal.pone.0272730 (PMC9362937; doi:10.1371/journal.pone.0272730)
Supplement: Attachment — Submitted filename: PLOS_simard_et_al_rebuttal_letter.docx [file pone.0272730.s003.docx]

**Editor comments:**

**The first and most problematic point is about data availability, as agreed by two reviewers. The main contribution of the paper is connected to the descriptive analysis of a very large database.  But the underlying data, classifications and indicators adopted are not appropriately described and released. The paper appears not to be compliant with data availability policy as stated here:** [**https://journals.plos.org/plosone/s/data-availability**](https://journals.plos.org/plosone/s/data-availability)**.**

**I think that there are two possible solutions to overcome this problem. The first one is to release anonymized raw data containing only the minimum set of information useful for replicating the analysis. For example, if you limit the set of metadata to an anonymous ID, country, type of open access, discipline/fields I think that this is not in conflict with the legal restrictions imposed by Clarivate. As an alternative or better as a complement to the raw data you should release the aggregated and reworked data.**

**I agree with Reviewer 2 that the presentation of data is not completely satisfactory, and I would suggest a better refinement of table and figures. Moreover, I think that the choice of omitting all computational details is a major shortcoming of the paper. For example, no clear definitions of fields/discipline classification of papers and normalizations are available for readers. Analogously readers are unaware of the classification of countries. I think that readers would benefit by a better documentation of your analysis.**

Answer: We have added some information about the computational details and updated all of the figures. The data used is now also available.

**Journal Requirements:**

**When submitting your revision, we need you to address these additional requirements.**

**1. Please ensure that your manuscript meets PLOS ONE's style requirements, including those for file naming. The PLOS ONE style templates can be found at**

[**https://journals.plos.org/plosone/s/file?id=wjVg/PLOSOne_formatting_sample_main_body.pdf**](https://journals.plos.org/plosone/s/file?id=wjVg/PLOSOne_formatting_sample_main_body.pdf) **and**

[**https://journals.plos.org/plosone/s/file?id=ba62/PLOSOne_formatting_sample_title_authors_affiliations.pdf**](https://journals.plos.org/plosone/s/file?id=ba62/PLOSOne_formatting_sample_title_authors_affiliations.pdf)

**2. Thank you for stating the following financial disclosure:**

**“YES - Vincent Larivière would like to acknowledge the Canada Research Chair program (grant 950-231768). Marc-André Simard would like to acknowledge the SSHRC Joseph Armand Bombardier Master’s Scholarship.”**

**Please state what role the funders took in the study.  If the funders had no role, please state: "The funders had no role in study design, data collection and analysis, decision to publish, or preparation of the manuscript."**

**If this statement is not correct you must amend it as needed.**

**Please include this amended Role of Funder statement in your cover letter; we will change the online submission form on your behalf.**

**3. Thank you for stating the following in your Competing Interests section:**

**“No”**

**Please complete your Competing Interests on the online submission form to state any Competing Interests. If you have no competing interests, please state "The authors have declared that no competing interests exist.", as detailed online in our guide for authors at** [**http://journals.plos.org/plosone/s/submit-now**](http://journals.plos.org/plosone/s/submit-now)

**This information should be included in your cover letter; we will change the online submission form on your behalf.**

**4. In your Data Availability statement, you have not specified where the minimal data set underlying the results described in your manuscript can be found. PLOS defines a study's minimal data set as the underlying data used to reach the conclusions drawn in the manuscript and any additional data required to replicate the reported study findings in their entirety. All PLOS journals require that the minimal data set be made fully available. For more information about our data policy, please see** [**http://journals.plos.org/plosone/s/data-availability**](http://journals.plos.org/plosone/s/data-availability)**.**

**Upon re-submitting your revised manuscript, please upload your study’s minimal underlying data set as either Supporting Information files or to a stable, public repository and include the relevant URLs, DOIs, or accession numbers within your revised cover letter. For a list of acceptable repositories, please see** [**http://journals.plos.org/plosone/s/data-availability#loc-recommended-repositories**](http://journals.plos.org/plosone/s/data-availability#loc-recommended-repositories)**. Any potentially identifying patient information must be fully anonymized.**

**Important: If there are ethical or legal restrictions to sharing your data publicly, please explain these restrictions in detail. Please see our guidelines for more information on what we consider unacceptable restrictions to publicly sharing data:** [**http://journals.plos.org/plosone/s/data-availability#loc-unacceptable-data-access-restrictions**](http://journals.plos.org/plosone/s/data-availability#loc-unacceptable-data-access-restrictions)**. Note that it is not acceptable for the authors to be the sole named individuals responsible for ensuring data access.**

**We will update your Data Availability statement to reflect the information you provide in your cover letter.**

Answer: We have shared a dataset that allows reproduction of the analyses.

**5. We note that Figures 1a, 2a, appendix1a and appendix1b in your submission contain [map/satellite] images which may be copyrighted. All PLOS content is published under the Creative Commons Attribution License (CC BY 4.0), which means that the manuscript, images, and Supporting Information files will be freely available online, and any third party is permitted to access, download, copy, distribute, and use these materials in any way, even commercially, with proper attribution. For these reasons, we cannot publish previously copyrighted maps or satellite images created using proprietary data, such as Google software (Google Maps, Street View, and Earth). For more information, see our copyright guidelines:** [**http://journals.plos.org/plosone/s/licenses-and-copyright**](http://journals.plos.org/plosone/s/licenses-and-copyright)**.**

**We require you to either (1) present written permission from the copyright holder to publish these figures specifically under the CC BY 4.0 license, or (2) remove the figures from your submission:**

**a. You may seek permission from the original copyright holder of Figures 1a, 2a, appendix1a and appendix1b to publish the content specifically under the CC BY 4.0 license.**

**We recommend that you contact the original copyright holder with the Content Permission Form (**[**http://journals.plos.org/plosone/s/file?id=7c09/content-permission-form.pdf**](http://journals.plos.org/plosone/s/file?id=7c09/content-permission-form.pdf)**) and the following text:**

**“I request permission for the open-access journal PLOS ONE to publish XXX under the Creative Commons Attribution License (CCAL) CC BY 4.0 (**[**http://creativecommons.org/licenses/by/4.0/**](http://creativecommons.org/licenses/by/4.0/)**). Please be aware that this license allows unrestricted use and distribution, even commercially, by third parties. Please reply and provide explicit written permission to publish XXX under a CC BY license and complete the attached form.”**

**Please upload the completed Content Permission Form or other proof of granted permissions as an "Other" file with your submission.**

**In the figure caption of the copyrighted figure, please include the following text: “Reprinted from [ref] under a CC BY license, with permission from [name of publisher], original copyright [original copyright year].”**

**b. If you are unable to obtain permission from the original copyright holder to publish these figures under the CC BY 4.0 license or if the copyright holder’s requirements are incompatible with the CC BY 4.0 license, please either i) remove the figure or ii) supply a replacement figure that complies with the CC BY 4.0 license. Please check copyright information on all replacement figures and update the figure caption with source information. If applicable, please specify in the figure caption text when a figure is similar but not identical to the original image and is therefore for illustrative purposes only.**

**The following resources for replacing copyrighted map figures may be helpful:**

**USGS National Map Viewer (public domain):** [**http://viewer.nationalmap.gov/viewer/**](http://viewer.nationalmap.gov/viewer/)

**The Gateway to Astronaut Photography of Earth (public domain):** [**http://eol.jsc.nasa.gov/sseop/clickmap/**](http://eol.jsc.nasa.gov/sseop/clickmap/)

**Maps at the CIA (public domain):** [**https://www.cia.gov/library/publications/the-world-factbook/index.html**](https://www.cia.gov/library/publications/the-world-factbook/index.html) **and** [**https://www.cia.gov/library/publications/cia-maps-publications/index.html**](https://www.cia.gov/library/publications/cia-maps-publications/index.html)

**NASA Earth Observatory (public domain):** [**http://earthobservatory.nasa.gov/**](http://earthobservatory.nasa.gov/)

**Landsat:** [**http://landsat.visibleearth.nasa.gov/**](http://landsat.visibleearth.nasa.gov/)

**USGS EROS (Earth Resources Observatory and Science (EROS) Center) (public domain):** [**http://eros.usgs.gov/#**](http://eros.usgs.gov/)

**Natural Earth (public domain):** [**http://www.naturalearthdata.com/**](http://www.naturalearthdata.com/)

Answer: The software we used for the maps (Tableau) uses maps from Open Street Map (see copyright notice in the bottom of our map) which has an open license:

<https://www.openstreetmap.org/>

**6. We noted in your submission details that a portion of your manuscript may have been presented or published elsewhere. Please clarify whether this publication was peer-reviewed and formally published. If this work was previously peer-reviewed and published, in the cover letter please provide the reason that this work does not constitute dual publication and should be included in the current manuscript.**

Answer: The original version was peer-reviewed and published as a part of the Proceedings of the 2021 International Conference on Scientometrics and Informetrics (ISSI, 2021) as a work in progress. The original version used older data and had less than 5 pages of content (can be found here: <https://tinyurl.com/MASOA>). For the new version, we updated the data and added more to every individual section.

**Comments**

**Reviewer #1:**

**Thank you for this timely and very interesting paper! Please see some comments and suggestions below.**

**I would add at low or no cost to this sentence in the Abstract: as well as the development of several new platforms that facilitate the publication of OA content at a low cost.**

Answer: Thank you for the suggestion, we modified the sentence as suggested.

**Background - Open access models - I am not sure ROAR data is up to date. I would use OpenDOAR for current repository statistics** [**https://v2.sherpa.ac.uk/opendoar/**](https://v2.sherpa.ac.uk/opendoar/) **- 5743 repositories as of October 2021** [**https://v2.sherpa.ac.uk/view/repository_visualisations/1.html**](https://v2.sherpa.ac.uk/view/repository_visualisations/1.html)**. And I would revise this sentence with data from OpenDOAR: "There are currently 4,607 repositories listed in the Registry of Open Access Repositories (ROAR), including 1,021 in North America (839 in the United States,), 1702 in Europe, 179 in Africa, 1021 in Asia, and 584 in South America."**

Answer: Thank you, we added the number of repositories reported in OpenDOAR alongside the updated numbers from the ROAR. We kept the ROAR data since the registry provides the numbers by continent, which we find useful in the context of this study.  The text now reads as follows:

As of November 6 2021, there were 5,764 and 4,629 repositories listed in the Directory of Open Access Repositories (OpenDOAR) and the Registry of Open Access Repositories (ROAR), respectively. The repositories are distributed as follows according to the ROAR data: Europe (36.8%), Asia (22.3%), North America (22.1%), South America (12.8%), Africa (3.9%), and Oceania (2.1%).

**I am not sure whether rumours is the accurate word in the sentence below, perhaps it could be replaced with discussions or something similar - In the United States, there have been rumors of a new OA initiative comparable to Plan S in Europe: this policy would make every…**

Answer: Thank you, we modified the sentence and used “discussions” instead.

**Perhaps it's also worth mentioning Redalyc** [**https://www.redalyc.org/**](https://www.redalyc.org/) **and AmeliCA** [**http://amelica.org/**](http://amelica.org/) **together with SciELO publishing platforms? And mention that SciELO collaborates with South Africa** [**http://www.scielo.org.za/**](http://www.scielo.org.za/)**? And what about repositories platforms - e.g. LA Referencia in Latin America** [**https://www.lareferencia.info/en/**](https://www.lareferencia.info/en/)**, CAS IR Grid in China** [**http://www.irgrid.ac.cn/**](http://www.irgrid.ac.cn/)**?**

Answer: Thank you. All of the suggestions were added.

**And I am not sure how accurate is this sentence in the Conclusion: This could be explained by the fact that APCs are mostly waived for Sub-Saharan countries (e.g. research4life countries). Perhaps you could add that APCs are mostly waived for low income countries and discounted for low-middle income countries? E.g. from Taylor and Francis** [**https://authorservices.taylorandfrancis.com/publishing-open-access/oa-funding-options/**](https://authorservices.taylorandfrancis.com/publishing-open-access/oa-funding-options/)**.**

Answer: We have added the income levels in order to make the statement more accurate. Thank you.

**I would be curious to see how many of OA journals in your study didn't charge APCs - APCs waivers have been mentioned as one of the reasons for OA publishing, but perhaps these were the journals that didn't charge APCs.**

**And perhaps the UNESCO reference below could be updated with the latest version of the Open Science Recommendation that governments will vote on in November:** [**https://unesdoc.unesco.org/ark:/48223/pf0000378381.locale=en**](https://unesdoc.unesco.org/ark:/48223/pf0000378381.locale=en)

**27. UNESCO. Towards a UNESCO Recommendation on Open Science [Internet]. 2020**

**[cited 2021 Jul 16]. Available from:**

[**https://en.unesco.org/sites/default/files/open_science_brochure_en.pdf**](https://en.unesco.org/sites/default/files/open_science_brochure_en.pdf)
Answer: Thank you. We have updated the reference and added a sentence on the official adoption of the Recommendation.

**Reviewer #2:**

**The manuscript is very clear and well presented and the data analysis has been done seriously. The corpus used is also clearly described and the literature review is quite well provided and informative. For all these qualities, it seems to me that the article deserves only minor revisions. That said, the article does have weaknesses and would benefit from substantial improvements. I provide in the following a set of comments made during the reading, which you will find in the uploaded version of the manuscript with comments. But before that, I think it is important to stress the two main weaknesses of the article:**

**- First, the result section is very brief and limited to simple comments of graphs and maps. All the non data-related material is in the long introduction and background and in the conclusion. This is perhaps a choice of presentation and I would be ready to accept it if it is justified, but from a rhetorical point of view, it seems to me that the contribution of the article would perhaps be more convincing with an enriched result section, why not with the help of some arguments and interpretations that are actually stated elsewhere in the article.**

Answer: Thank you for the comment. Indeed, we did not comment on the results within the results section on purpose. We chose to describe (results) and then discuss in the now renamed discussion and conclusion section.

**- Secondly, the maps and graphs suffer from many flaws and need to be reworked. This is all the more inconvenient as they occupy a central place in the result section and are therefore essential elements in the purpose and contribution of this article.**

Answer: The file conversion process had a very negative effect on the resolution of the files. When we noticed this, the submission had already been sent. New higher definition versions of the figures have been added. Thank you.

**Finally, the raw data cannot be shared because of the agreement with Clarivates, but the aggregated and reworked data (Z-score at country level) are certainly not affected by this same restriction and would benefit from being openly shared.**

Answer: We prepared a dataset that can be shared. Thank you.

**In what follows, I report the comments made in a linear way when reading the article and which you can also find in the commented version of the manuscript:**

**Page 5: As you processed the data and have indicators at the global scale, I believe you could share the Z-scores you computed at the country level**

Answer: The Z-scores will be a part of the new dataset. Thank you.

**Page 10 :**

**- "with the creation of a dozen more journals" --> "with the creation of a dozen more electronic journals"**

Answer: Added, thank you.

**- You should introduce the plan of the article at the end of the introduction (before the background section)**

**Page 13 :missing word before "at": "In terms of OA usage by different at the country level"**

Answer: Thank you, we fixed the sentence.

**Page 14, last paragraph:Note that the result of the paper by Iyandemye and Thomas is already cited in a previous paragraph.**

Answer: Thank you, we modified the sentence to only refer to the part of the Iyandemye and Thomas results that relate to this paragraph, and thus removed the repetition.

**Page 16, first paragraph: Note that the NIH policy is already mentioned earlier in the paper.**

Answer: Thank you, we removed the second mention of the NIH policy.

**Page 16, first paragraph: "has contributed to the expensive of PMC" --> "to the expansion of PMC"**

Answer: Thank you, we made the correction.

**Page 18, first paragraph: Given that the number of journals with DOIs may not be very evenly distributed around the world, I think that ignoring articles without DOIs could skew your analysis. If you think so too, I think it would be worth mentioning this limitation.**

Answer: Thank you. We have added a sentence to mention this limitation.

**Page 18, first paragraph: "This data indicates whether the paper is published in OA or not, and in the OA category it belongs to" --> "and in the OA category it belongs to" (useless "in")**

Answer: Thank you, we made the correction.

**Page 18, second paragraph, first sentence: useless "that"**

Answer: Thank you, we made the correction.

**Page 18, last paragraph: less OA papers in the Humanities than in Medecine --> This result must be interpreted with caution since the type of social sciences and humanities journals indexed on the Web of Science are mostly owned by for profit publishers such as Wiley. In other words, don't you think the WoS coverage bias can affect this observation?**

Answer: The data source is definitely one of the limits of this study. We have added a sentence to reflect this situation in the discussion. Thank you.

**Page 20, first sentence: "Figures 1 and 2 reveal that countries mostly cite OA more often than they publish in OA." --> You latter show that this really depends on the countries. Therefore this first sentence should be rewritten. If you really want to state that "most countries cite OA more often than they publish in OA", then I advise you to use a global statistics i.e. the % of countries citing OA more often than they publish in OA.**

Answer: We removed this sentence as it was indeed problematic. Thank you.

**Page 20, last sentence: It would be clearer for the reader if you specify in parenthesis after both indicator what is exactly the variable that you used (I believe it is the z-score mentioned in the method section but helping the reader would be useful here).**

Answer: We have added the % of references and OA publications that the text referred to. Thank you.

**Page 21, first sentence: The relation is not that strong at the global level indeed, justifying to look at the divergence between groups of countries regarding this statistical relation as you have done below.**

**Page 22: "This could be explained by the fact that APCs are mostly waived for Sub-Saharan countries (e.g. research4life countries) but are not or only partially waived for most Middle Eastern and Asian countries" --> Therefore, It could have been interesting to consider groups of countries according to these differences instead of using the world bank's classification**

Answer: That is a good point. However, we felt that using the World Bank’s classification gave us a good “frame” for this. We also wanted our results to be comparable to those of Evans and Reimer (2009) who also used the World Bank’s classification for countries.

**Page 23: "This may imply that even in the richest countries on the planet, researchers may still struggle to pay APCs." --> Or be opposed to them?**

Answer: Or both!  Thank you, we added this to the paper.

**Page 24: "Despite the existence of geological tensions" --> "geopolitical" instead of "geological"**

Answer: Thank you, we made the correction.

**Page 31: Figure 1: This map is blur and the projection must be changed (the mercator projection is only relevant when considering sea travels since it preserves navigation angles) :** [**https://www.britannica.com/science/Mercator-projection**](https://www.britannica.com/science/Mercator-projection)

Answer: The software we used to generate the maps (Tableau) does not seem to allow different types of projection.

**Page 32: The legend should be on the map and a precision on the unit of measure should be added**

Answer: We fixed the map and the legend, thank you.

**Page 33 and 34: Figure 2: same remarks as for Figure 1a and Figure 1b**

Answer: We fixed the map and the legend, thank you

**Page 35, Figure 3: This graphic could be much more interesting and self-explanatory in labelling the nodes and in adding the formula of the regression line in a box. See for instance with R:** [**https://www.r-graph-gallery.com/web-scatterplot-corruption-and-human-development.html**](https://www.r-graph-gallery.com/web-scatterplot-corruption-and-human-development.html) **and:** [**https://rpkgs.datanovia.com/ggpubr/reference/stat_regline_equation.html**](https://rpkgs.datanovia.com/ggpubr/reference/stat_regline_equation.html)

Answer: Thank you, we redid the figures in R to make them nicer and clearer.

**Page 36, Figure 4: Same remark as for the previous plot, the quality of these plots is very low. In addition, the dots are too big which creates a lot of superpositions.**

Answer: Thank you, we redid the figures in R to make them nicer and clearer.

**Page 37, Figure 5: Blur but fine**

Answer: We increased the resolution of the figure to resolve this. Thank you.

**Page 38, appendix: The projection is incorrect and the legend is not explanatory (the unit of measure is missing)**

Answer: We fixed the maps and the legends, thank you. However, as previously stated, the software we used (Tableau) does not seem to allow the use of different projections.
