# Supplementary material for: National differences in dissemination and use of open access literature
Source: PLoS One. 2022 Aug 9;17(8):e0272730. doi: 10.1371/journal.pone.0272730 (PMC9362937; doi:10.1371/journal.pone.0272730)
Supplement: Attachment — Submitted filename: PLOS_simard_et_al_rebuttal_letter_second_round.docx [file pone.0272730.s004.docx]

**Letter to reviewers:**

Please use the space provided to explain your answers to the questions above. You may also include additional comments for the author, including concerns about dual publication, research ethics, or publication ethics. (Please upload your review as an attachment if it exceeds 20,000 characters)

Reviewer #1: (No Response)

Reviewer #2: I would like to thank the authors for the new version of their article and especially for the open access and sharing of the data. This gives a real added value to their contribution.

**Answer: Thank you!**

Despite the changes made, I have identified a number of shortcomings in the revised text that will need to be corrected before the article can be published.

1. Contrary to what the authors have indicated, the repetition regarding the NIH policy is still present in the current version: to avoid this repetition, one option is to move the paragraph on NIH that is in the “OA models” section to the “initiatives and platforms” section (where the NIH policy is introduced).

**Answer: Our apologies for the confusion about that part. We have removed it from the paper. Thank you!**

2. Some references are not properly introduced, which can make them seem irrelevant or confusing (they are listed below).

**Answer: We have fixed the references based on your suggestions. Thank you!**

3. Contrary to what was requested, the maps have not been redone. To solve this issue, we provide the authors with a script and a figure adapted from their data that they can reuse to generate Figure 1 and 2. We show in this script that it is possible to make a discretization of the z-score variable rather than representing it as a continuous variable. This approach allows to obtain a clearer figure and to better manage extreme values such as the score of Equatorial Guinea (in the authors' version, it seems that it has been removed (as the max is 2 on the legend) which is problematic). We also note that the data is missing for Greenland (given that there is a university in Greenland, is this due to missing data or an omission?)

**Answer: We have redone the maps based on your suggestions and code provided. Thank you very much for your kindness.**

4. Finally, we have a suggestion to make to improve the readability of the 1st part. As the first part of the article lists a large number of facts that made it possible to reconstruct a chronology of the development of Open Access: on re-reading, we think that it would be helpful to add a timeline or a summary listing the main events mentioned in the text in chronological order. This will facilitate the reading of the text. If the authors do not wish to add a new figure in the body of the text, they can add this timeline in an appendix. As such, it will be an additional result.

**Answer: We thank the reviewer for this valuable suggestion. However, we chose not to include a timeline in the article because of time constraints.**

In the following we list the changes requested and the problems identified as they appear in the text:

Page 1: Replace "Psycholoquy" by "Psychology"

**Answer: Psycholoquy is the name of an early OA journal.**

Page 3: Replace "Bronze OA refers to publications that are openly directly" by "Bronze OA refers to publications that are open directly"

**Answer: We have changed the sentence. Thank you!**

Page 4: Miguel et al. is cited (between Pinowar et al and Basson et al) whereas it seems that contrary to these other references Miguel et al concentrate on the literature dealing with OA which is another matter and should be introduced as such.

**Answer: The reference has been moved to the next paragraph and reintroduced in a way that highlights its contribution (countries who contribute to OA as a topic).**

Page 4: Replace "their effects of the measurement of open access" by "their effects on the measurement of open access"

**Answer: We have changed the sentence. Thank you!**

Page 5: missing dot before reference number 8 (later in the text, we found that there are sometimes missing spaces before reference numbers: eg. before reference 21)

**Answer: Thank you for pointing that out. We believe that they have all been fixed now.**

In the “Initiative and Platforms” section: you should create a new paragraph for each geographic area: “In Europe”, “In Canada”, “In the US”, “In South America”, “In Asia”, “In Africa”. It will improve the clarity of this section.

**Answer: New paragraphs and titles have been added. Thank you!**

Page 7, in the paragraph about Canada: The reference to the OJS system (32) seems a bit out of context as the link between this system and OA is not explicated

**Answer: We removed the reference to the OJS as it is not necessarily an OA focused initiative. Thank you.**

Page 10: The acronym “BOAI” is mentioned whereas it has never been introduced before

**Answer: We added the acronym to the first mention of the BOAI. Thank you!**

Results section, 1st sentence: Replace "Tables 1 and 2 presents" by "Tables 1 and 2 present"

**Answer: Done, thank you!**

Table 1 caption: missing parenthesis

**Answer: Done, thank you!**

Figure 5: on the left part, green is in yellow and gold is in green

**Answer: Done, thank you!**

Figure 1 and 2 + Appendix: the maps should be redraw with the help of the provided script (it is possible to change the colours if you wish to, and, if necessary, to add an intermediary class around zero to represent countries with z-scores equal to the world average).

**Answer: We used your script to make the new figures. Thank you so much.**
